# Emergency care research as a global health priority: key scientific opportunities and challenges

**DOI:** 10.1136/bmjgh-2019-001486

**Published:** 2019-07-29

**Authors:** Junaid Razzak, Blythe Beecroft, Jeremy Brown, Stephen Hargarten, Nalini Anand

**Affiliations:** 1 Department of Emergency Medicine, Johns Hopkins University, Baltimore, Maryland, USA; 2 Center for Global Health Studies, John E Fogarty International Center, Bethesda, Maryland, USA; 3 Office of Emergency Care Research, National Institutes of Health, Bethesda, Maryland, USA; 4 Department of Emergency Medicine, Medical College of Wisconsin, Milwaukee, Wisconsin, USA

**Keywords:** injury

## Abstract

Quality emergency medical care is critical to reducing the burden of disease in low-income and middle-income countries (LMICs) and protecting the health of populations during disasters and epidemics. However, conducting research in emergency care settings in LMIC settings entails unique methodological and operational challenges. Therefore, new approaches and strategies that address these challenges need to be developed and will require increased attention from scientists, academic institutions and the global health research funding community. Research priorities to address emergency care in LMICs have also not been well defined, resulting in limited research output from LMICs. This manuscript frames the efforts of four multidisciplinary working groups, which were established under the auspices of the Fogarty International Center as part of the Collaborative on Enhancing Emergency Care Research in LMICs and serves as an introduction to this series, which identifies challenges and solutions in the context of emergency care research in LMICs. The objective of this introductory paper is to articulate the need for emergency care research in LMICs and underscore its future promise. We present public health arguments for greater investment in emergency care research, identify barriers to develop and conduct research, and present a list of research priorities for community organizations, academic institutions and funding agencies. We conclude that advances in emergency care research will be critical to achieve national and global health targets, such as the Sustainable Development Goals (SDGs), and to ensure that evidence informs how such research is best conducted.

**Summary box**

- Relatively low research investments and lack of expertise in emergency care research have resulted in considerable disparities between the burden of emergency diseases and research output.
- Despite challenges, there are multiple compelling reasons to conduct and invest in emergency care research and research capacity building in low-income and middle-income countries.
- The Collaborative on Enhancing Emergency Care Research in LMICs effort recommends: strengthening emergency care research capacity, providing opportunities for collaboration and networking, increasing support for research and training from the research funding community and philanthropic organisations, standardising definitions of outcomes and exploring the use of technology for emergency care research.

## Introduction

**Figure 1:**
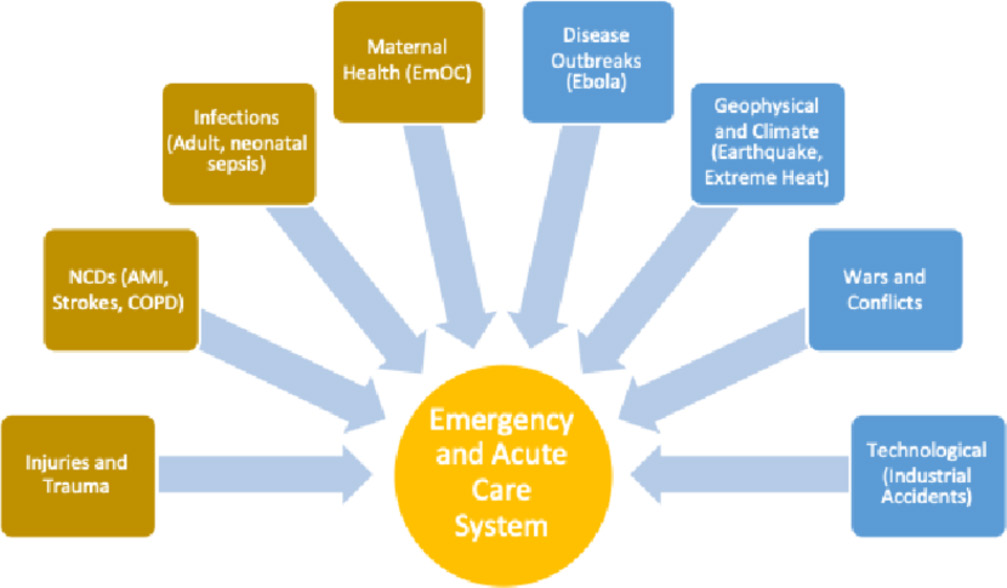
Scope of emergency care.

Approximately half of the total burden of diseases in low-income and middle-income countries (LMICs) is caused by time-sensitive emergency or acute illnesses and injuries.^1–3^ According to the Disease Control Priorities Project (DCP2), as much as 45% of the disease burden in LMICs can be at least partially addressed by an effective and functional emergency care system. Five of the most frequent causes of death in LMICs—ischaemic heart disease, stroke, lower respiratory infections, chronic obstructive pulmonary disease (COPD) and diarrheal diseases, primarily present as an emergency, have time-sensitive treatments and show improved outcomes with quality acute care. The same is true for many causes of maternal and neonatal deaths, as well as injuries.^4^ A recent analysis has shown a fivefold difference between the prevalence rates of emergency, time-sensitive diseases in high-income versus low-income countries.^2^ Effective emergency medical care can serve as a health system intervention impacting health outcomes for a significant percentage of people dying or suffering disabilities in LMICs. Similarly, emergency medical care is a critical healthcare system during public health emergencies caused by various forms of humanitarian crises (figure 1). The recent Ebola outbreak in West Africa highlighted the need to strengthen acute and emergency care systems in LMICs, while benefiting the global community. For purposes of this Supplement and the Collaborative on Enhancing Emergency Care Research in LMICs (CLEER), we focus on emergency care at the individual level.

## Definition of emergency care

**Figure 2:**
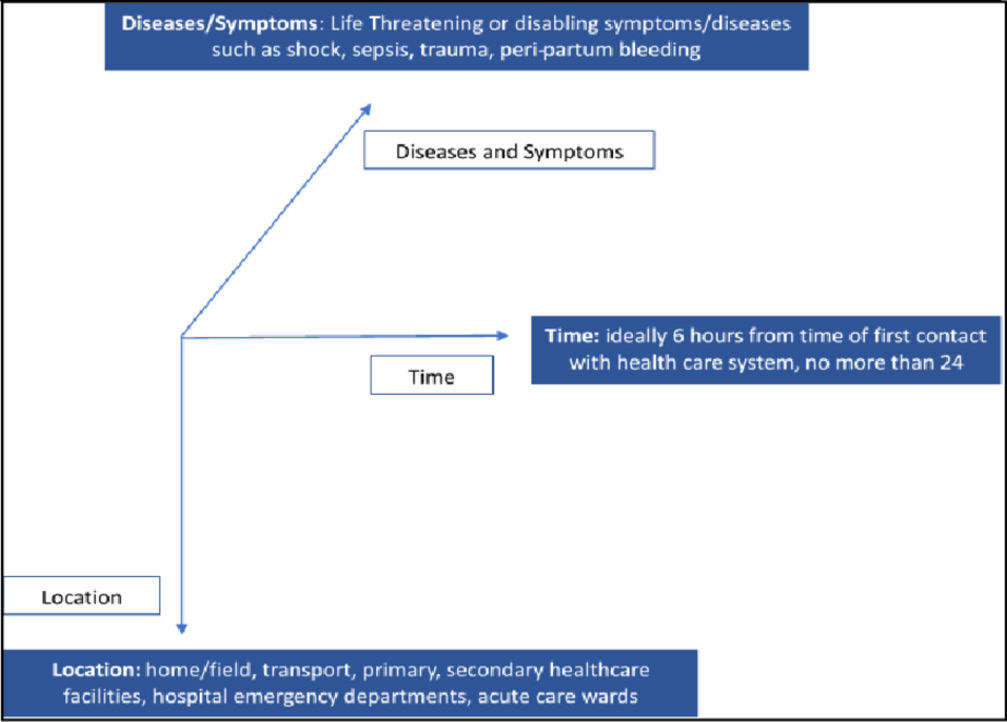
Definition of emergency care.

The term emergency care means different things in different contexts. As shown in figure 2, our definition encompasses the three essential components of emergency care, that is, time, location and diagnoses/symptoms.

For this series, we have limited the timeframe to care provided to a patient with acute, potentially life-threatening/disabling symptoms within the first 6 hours of contact with a health facility/provider. The time period of 6 hours captures treatment strategies and critical decisions for many conditions, such as sepsis, myocardial infarction, strokes, acute injury and maternal haemorrhage. The second component, location, generally includes emergency departments, ambulances, urgent care centres, and so on. In places where formal ambulances and emergency departments do not exist or have variable definitions, we define location as the location of the patient, or wherever initial life-saving care can be provided. The third component is the disease/symptom dimension, which includes a considerable number of conditions that can be classified as emergency or conditions, including injuries/trauma, myocardial infarction, stroke, COPD, asthma, allergic reactions, sepsis, maternal haemorrhage and pneumonia. Often early in emergency care, diagnoses are unclear and a patient’s journey starts with certain key symptoms, such as chest pain, severe headache, loss of consciousness or focal weakness. Therefore, the working definition of emergency care combines the time dimension of the first 6 hours with patients presenting potentially life-threatening symptoms or diagnosis anywhere in the healthcare system.

## Public health imperative

Strong emergency care systems based on robust evidence are critical to advancing global health. However, conducting research in the context of emergency care involves many unique challenges. This Supplement lays out an agenda for tackling these challenges. There are multiple compelling reasons to invest in emergency care research and research capacity building in LMICs.

1. Burden of emergency diseases: Emergency, time-sensitive illnesses contribute to the majority of the disease burden in LMICs. According to Chang *et al*, 60% of disability-adjusted life years in LMICs are caused by emergency medical conditions.^4^
2. Cost-effectiveness of emergency care interventions: Data on the cost-effectiveness of major public health interventions identified emergency medical interventions as some of the most cost-effective. For example, DCP defined the availability of volunteer prehospital care/ambulance services as the second most cost-effective public health intervention. Similarly, aspirin for myocardial infarctions and formal paramedic-run emergency medical care were identified as 6th and 21st most cost-effective interventions.^1^
3. Emergency care systems are sources of important data and can help to better define the epidemiology for acute diseases, such as injuries, myocardial infarctions, cerebrovascular diseases and infections, and serve as surveillance systems during epidemics, such as Ebola, influenza and Zika. The acute care setting can also serve as a site for clinical trials for acute medical and surgical interventions.
4. Effective emergency care systems can serve as settings for public health interventions for difficult to reach populations: There are successful examples from high-income countries (HICs) on the role of emergency care systems in health promotion and disease prevention, such as smoking, drug and alcohol dependence, domestic violence, self-harm, hypertension screening and referral, and injury prevention.^5^
5. Global health security: The concept of ‘health security’, or the protection from health threats, has recently been recognised as one of the most critical international security issues—particularly in light of the recent Ebola and Zika virus outbreaks.^6^ In response to these increasing global health security concerns, efforts to build capacity in infectious disease and all-hazards disaster preparedness and response among developing and developed countries alike is an urgent priority.
6. Need for context-specific interventions in LMICs: Differences in disease profile and patient characteristics in LMICs require that interventions are properly tailored to specific contexts and not automatically transferred from HICs to LMICs. For example, early data on resuscitation have highlighted crucial differences in emergency patients in some LMICs, which require a different approach to resuscitation. A study by Maitland *et al* showed the harmful effects of implementing fluid resuscitation guidelines developed in the USA for treating children in Sub-Saharan Africa.^7^
7. Global health and development priorities: The United Nations SDGs expect countries to achieve ambitious targets to reduce mortality and morbidity due to non-communicable diseases, road traffic injuries, and newborn and maternal deaths, which will remain unachievable without strong emergency care systems.^8^ Table 1 presents the individual SDG targets and their relationships to emergency care.

**Table 1:** Sustainable development goals and emergency care

SDG target	Community emergency care	Transport emergency care	First-level facility emergency care	Hospital-based emergency care
1. Maternal mortality	✓	✓	✓	✓
2. Child mortality	✓	✓	✓	✓
3. AIDS/TB/malaria	✓	✓	✓	✓
4. Non-communicable diseases	✓	✓	✓	✓
5. Substance abuse	✓	✓	✓	✓
6. Road traffic injuries	✓	✓	✓	✓
7. Sexual and reproductive health			✓	
8. Climate/environment			✓	✓
9. Essential medicines/vaccines	Defining and advocating for essential ‘life-saving’ medicines			
10. Universal health coverage	Emergency care should be the key component of ‘essential’ health services			
11. Health workforce	Thinking outside the box about healthcare workforce development			
12. Health crisis	Disaster preparedness and response			

SDG, sustainable development goal; TB, tuberculosis.

## Investments in emergency care research

The National Institutes of Health (NIH) is just one of many funding organisations that share responsibility for supporting critical global health research priorities, but it is nonetheless informative to examine NIH investments in emergency care research. In general, NIH spends about 0.7% of its research budget on new research projects in emergency care.^9^ In 2014 that amounted to US$25.5 million, but a further US$34.6 million supported ongoing (rather than new) emergency care research.^9^ Among six specialties reviewed (emergency medicine, family medicine, internal medicine, obstetrics-gynaecology, paediatrics, and psychiatry and surgery), emergency medicine was the fifth-lowest in terms of funding per resident in training.^10^ An unpublished review of a decade of funding to six specialties revealed that emergency medicine faculty (which is a subset of all those performing research in emergency *care*) had the lowest number of NIH grants funded over the 10-year period.^11^

Research capacity in emergency care in LMICs is limited. While there has been a significant increase in the number of clinical training programmes in Africa, Latin America and Asia, the focus of these programmes is service delivery and a few programmes have an academic focus or support. Notably, NIH has funded research training programmes, such as the Fogarty Global Injury and Trauma Research Training Program, to support emergency medical care research capacity building, among other areas.^12^ This is a relatively small programme and unique effort, given the magnitude of impact emergency care can have on the public health outcomes.

In terms of research output, a PubMed search for the term ‘emergency care’ identified a total of 169 315 articles of which 8064 were identified as ‘clinical trials’ during a 10-year period of 2008–2018. When the search was limited to publications with authors from the current low-income countries (Cambodia, Chad, South Sudan, Tanzania, Zimbabwe, Comoros, Haiti, Benin, Nepal, Mali, Sierra Leone, Burkina Faso, Afghanistan, Uganda, Rwanda, Mozambique, Togo, Guinea-Bissau, North Korea, Ethiopia, Eritrea, Guinea, Gambia, Madagascar, Niger, Democratic Republic of Congo, Liberia, Central African Republic, Burundi, Malawi and Somalia), the total number of publications went down to 1344 (0.79%). Of these, only 44 were clinical trials (0.54%).

## Research challenges in emergency care in LMICs

Research in the acute care context in LMICs is challenging for a variety of reasons. Some of these challenges are described below:

1. Defining and capturing the population of interest: Medical emergencies can happen at any location and because emergency care is provided at any location—from home to transport to the different levels of health facilities—it is often difficult to consistently capture all patients presenting with diseases or symptoms of interest to the researchers. Also, at least initially, patients present with symptoms and not diagnoses; since most of the health research is funded by and focused on diagnoses, this adds another level of complexity. The diagnostic certainty is further compromised in low-resource settings due to the limitation of diagnostic capabilities and trained personnel in emergency care settings.
2. Defining interventions and outcomes: Interventions are relatively easy to define in emergency care, while outcomes are often difficult to frame. As described in the manuscripts on emergency care clinical research and emergency health systems research in this Supplement, longer-term and more meaningful outcomes are often not available for many emergency care interventions. Outcomes are frequently defined by results in the first few hours of presentation and include either mortality outcomes or health services outcomes, such as admission versus discharge from the hospital. While emergency care interventions are short-term, long-term outcomes need to be captured as well.^13^
3. Study design and data collection: There are clear challenges in data collection, data analysis and comparability of research findings.^14^ Data collection is impacted by the acute, often life-threatening nature of disease presentation, the time sensitivity of interventions, the dynamic and volatile research environment and the over-burdened infrastructure. Data analysis is affected by symptom-based diagnosis, the availability of triage information and concerns about confounders in the environment. Data comparison presents challenges due to a lack of standardised data definitions.^13^ In most low-resource settings, emergency care data capture is not a priority for the already stretched emergency care system. Clinical information is often captured through a paper-based data system and is rarely archived unless patients are admitted to the hospital. In one review, only 10% of emergency department-based studies from LMICs used a specific diagnosis coding system.^15^
4. Ethical issues: Privacy, community engagement, fair participant selection and the ability to give and obtain quality informed consent in emergency care settings can be difficult. As discussed in the Ethics paper in this series, these challenges are intensified in LMICs by multiple factors including weak health infrastructure, high patient volumes, overworked providers and especially vulnerable populations.^16^ Gaps remain regarding ethical guidelines and regulations for research and best practices.
5. Research capacity and research environment: Few academic departments in LMICs focus on emergency care. Emergency care remains largely a hospital service with no academic home in medical schools and universities.

## Collaborative on Enhancing Emergency Care Research in LMICs

In July 2017, the Center for Global Health Studies at the Fogarty International Center at the NIH, convened a group of researchers with expertise in emergency care in LMICs to explore pressing research priorities in emergency care in LMICs, as part of the CLEER. The 39 expert participants were accepted from a pool of applicants and subsequently divided into four working groups of 7–12 focusing on (1) ethics, (2) surveillance and registries, (3) health systems and (4) clinical research. The participants represented 16 different countries and were mainly emergency medicine clinicians in both HICs and LMICs, joined by a few bioethicists and paediatricians. The group met physically at NIH for 2 days in July 2017 and then continued to teleconference several times over the next year in the four separate working groups.

The goal of CLEER is to promote research that improves outcomes for patients and populations with acute life-threatening or disabling conditions, focused on the care provided in the first minutes to hours of illness or injury. The groups’ mandate was to (1) identify important research gaps and critical research questions based on the level of the current evidence (table 2) and (2) explore the methodological issues in answering some of these questions with a focus on population, design, outcomes, ethics and research environment and support structure.

**Table 2:** Key research gaps and questions

Data and data systems	How can data from vertical programmes and non-health data be incorporated into emergency care surveillance?
	What are the validity, reliability and utility of various surveillance instruments used in emergency care settings?
	How do we predict the population-level burden of acute illnesses and injuries using data obtained through emergency care system surveillance?
	How do we accurately and reliably identify epidemiological changes in the health of communities through data obtained from emergency care system surveillance?
	What data obtained through emergency department routine surveillance can help with identifying infectious disease outbreaks in LMICs?
	How do we use emergency care surveillance data to better characterise the prevalence of non-communicable diseases (such as DM and HTN) in otherwise healthy patients and what mechanism would improve long-term care of such patients?
Quality and access to emergency care and clinical interventions for key diseases	What is the epidemiology of emergency diseases in low-resource settings? Can presenting symptoms and syndromic presentations be used to define disease epidemiology when time and/or resources to make final diagnoses are not always available?
	What are the measures of access to emergency care? What is the level of access of population in LMICs to quality emergency care?
	How can quality of emergency care be measured in low-resource settings? What interventions can be developed to improve the quality of emergency care? Which tools developed in high-resource settings are applicable in low-resource settings?
	Which component(s) of the emergency care system either individually or in combination are most effective at improving patient outcomes and decreasing risk of death and disability?
	How to identify, triage and treat patients with emergency conditions, such as sepsis, injury, etc, using vital signs and simple clinical assessments and other low-cost technologies, such as oxygen saturation?
	How do we strengthen risk assessment and engage acutely sick patients and their families in decision making in low-resource, low-health literacy settings?
Emergency health economics	What is the economic value of emergency care? What are the economic benefits of emergency care interventions?
	How do various methods of healthcare financing impact financial protection from emergency diseases?
Emergency care research ethics	What international-specific and country-specific guidelines could help researchers and research ethics committees navigate ethical and regulatory issues distinctive of emergency care research?

LMICs, low-income and middle-income countries.

CLEER was not intended to duplicate existing work focused on strengthening emergency care research. Previous efforts to examine how to best strengthen research in acute care settings and have largely been limited to the USA and Europe, and thus have not addressed the specific challenges for acute care research in LMICs. The Society of Academic Emergency Medicine did call a consensus meeting in 2013 to identify gaps and develop a research agenda for acute care services delivery in LMICs. The conference and resulting publication provided a broad research agenda with specific questions related to strengthening and sustaining effective acute care systems.^16^ CLEER has built on this agenda, focusing on four distinct aspects of research conducted in emergency care settings: emergency care surveillance and emergency care registries, clinical emergency care research, establishing economic value of emergency care and research on emergency care system and emergency care research ethics.

## Recommendations

### Strengthening emergency care research capacity in LMICs

Almost all subgroups of CLEER highlight the need for building the individual and institutional capacity for research in emergency care in LMICs. There have been few successful models, such as the Fogarty International Center’s D43 training grant mechanism, which has helped emergency care research capacity in South Asia and Africa. Similarly, the Medical Education Partnership Initiative targeted academic capacity in emergency medicine in a few institutions in Sub-Saharan Africa. More targeted programmes on emergency care research would provide support and incentives for research institutions in HICs to collaborate and support emergency care researchers in LMICs.

### Create opportunities for collaboration and networking

There are several professional societies and groups with an interest in various aspects of emergency care. Besides the societies in emergency medicine, national and international associations of paediatrics, surgery, infectious diseases and critical care include emergency care as part of their larger portfolio. However, CLEER is the first multidisciplinary group specifically targeting emergency care research in LMICs. If formalised, such groups can provide the necessary structure for global collaboration and networking.

### Funding and support for emergency care research

Challenges with respect to funding include the fact that disease-focused requests for proposals often do not clearly fit the syndrome/symptom-based patient presentations in the emergency care setting. Also, ethical and logistical challenges make these grants less competitive in contrast to cleaner, stable research environments in non-emergency settings. Support specifically for research in emergency care settings would enable the science to grow and research methodologies in emergency medicine to be developed such that emergency medicine researchers can compete with more established fields of medicine and public health.

### Standardised credible outcome measures

All of the CLEER subgroups highlight the difficulty in defining the input and outcome indicators for emergency care—at the individual, population and system level. Further work needs to be encouraged by the specialty societies to develop credit indicators, their definitions and method of measurement.

### Explore use of technology for research

The surveillance and clinical research groups of CLEER highlight the need for better use of technology, especially mobile technology, for data collection as well as potential clinical interventions.

## Conclusion

Emergency care is a critical entryway into the healthcare system and a key determinant of individual and population health, especially in LMICs. In this Supplement, experts articulate research gaps, needs and opportunities, while presenting a way forward with some innovative solutions. Specifically, the CLEER effort calls for strengthening emergency care research capacity, providing opportunities for collaboration and networking, increasing support for research and training from the research funding community and philanthropic organisations, standardising definitions of outcomes and exploring the use of technology for emergency care research.
